# Rolling bearing fault diagnosis based on acoustic-vibration data fusion and mode decomposition combined with the crested porcupine optimization algorithm

**DOI:** 10.1016/j.heliyon.2024.e40351

**Published:** 2024-11-12

**Authors:** Minyuan Jiang, Min Luo, Chaoyong Zhang, Min Shu, Guohao Sun

**Affiliations:** a School of Electrical and Information Engineering, Hubei University of Automotive Technology, Shiyan, 442002, China; b School of Mechanical Science and Engineering, Huazhong University of Science and Technology, Wuhan, 430074, China; c Hangzhou Yineng Electric Power Technology Co., Ltd, Hangzhou, 310000, China

**Keywords:** Variational mode decomposition, Crested porcupine optimization algorithm, Multi-modal data fusion, Bearing fault diagnosis

## Abstract

Variational Mode Decomposition (VMD) is extensively utilized in the domain of rotating machinery fault diagnosis. Nevertheless, the reliance on empirical parameter tuning and the limitations inherent in traditional single-signal (either vibration signals or acoustic signals) fault diagnosis methods present substantial challenges. These include incomplete information and high sensitivity to noise. To address these shortcomings, a new method is introduced that integrates multi-modal sensor data and implements an adaptive VMD approach, enhanced through the Crested Porcupine Optimization Algorithm (CPO) to automatically optimize the key parameter. Then, uses optimized VMD to decompose the time series into intrinsic mode functions of different frequencies to capture the multi-scale characteristics of data. Finally, the decomposed acoustic-vibro signal components are fed into the CNN-BiGRU-AT network, which learns the spatio-temporal dependencies to enhance the accuracy of fault classification. The experimental results conducted across diverse high-noise environments demonstrate that this method’s superior noise robustness compared to single-modality sensor approaches， highlighting the method's potential for machinery fault detection in noisy conditions.

## 1 Introduction

In the rapidly evolving industrial era, the pervasive use of mechanical equipment has penetrated critical sectors such as rail transport, manufacturing, and aerospace. Bearings, as pivotal in the operational integrity components of rotating machinery systems. Meanwhile, bearings are also among the components most susceptible to failures [1]. Consequently, timely and effective diagnosis of bearing faults has become an indispensable aspect of rotating machinery maintenance. This field has attracted sustained and extensive research attention over the past decades.

The health assessment of bearings can utilize a multitude of sensing technologies and signal processing methods, with various types of sensors capable of capturing fault characteristics. Commonly employed sensors include those for vibration, acoustic signals, acoustic emission, temperature measurements, and induction-based monitoring [[2], [3], [4]]. At present, most fault diagnosis methods are still single-type signal diagnosis. There are limitations such as limited information and noise interference. It is an important research direction in the field of fault diagnosis to combine different modal data to realize equipment anomaly recognition. On the one hand, the modal combination method considers the correlation between different data, and on the other hand, multiple data measurement points can cover a larger monitoring range of equipment. However, how to fuse different scale data from multiple types of sensors remains an important challenge. Wan et al. fused multiple types of data and extracted deeper features with neural network, highlighting the effectiveness of fault diagnosis network fusion of multiple types of data [5]. Wang et al. studied a bearing fault diagnosis method based on the fusion of vibration and sound signals, using a one-dimensional convolutional neural network (1D-CNN) for signal processing and fault identification [6]. Several studies have integrated acoustic and vibration signals of electrical equipment to realize state perception or fault diagnosis, and adopts the method of original data feature extraction to improve the fault identification effect of machine learning model [[7], [8], [9]].

Signal feature extraction and pattern recognition are the essence of bearing fault diagnosis algorithms, which can be accomplished using various techniques such as Fast Fourier Transform (FFT) [10], wavelet transform [11], Hilbert-Huang Transform (HHT) [12], Empirical Mode Decomposition (EMD) [13], Ensemble EMD (EEMD) [14], and Variational Mode Decomposition (VMD) [15]. FFT excels in extraction efficiency, although it performs poorly under non-linear and non-steady conditions. In contrast, wavelet transform is tailored for multi-resolution analysis, but is less effective with amplitude-modulated signals due to the choice of basis functions. EMD serves as a versatile bi-dimensional filter for analyzing complex signals but struggles with noise and sampling errors in experimental settings. VMD, using a non-recursive approach, mitigates errors and mode mixing effectively. However, the application of VMD requires pre-setting the number of modes K and the penalty factor α, where choosing suitable parameters significantly impacts the signal decomposition outcome. This necessitates an automatic optimization algorithm to fine-tune VMD's critical parameters, aiming to enhance its adaptability and the accuracy of signal decomposition, thereby enabling more precise extraction of features from fault signals to provide a more reliable data foundation for pattern recognition.

In the literature, various novel methods have been proposed to address the challenges associated with setting VMD parameters. The Whale Algorithm has been employed to optimize penalty factors for each mode, effectively filtering modal components related to bearing faults and extracting fault characteristics through envelope demodulation techniques [16]. Li et al. introduced a new method based on the envelope kurtosis maximization criterion to optimally determine the modal number, which effectively solved the problem of predetermining the VMD modal number [17]. Mohanty et al. compared vibration and acoustic signals at variable speeds and proposed a new Hurst index for VMD and EMD signals to identify actual modes describing fault frequencies [18]. Wang et al. developed an adaptive method for the determination of the number of modes on the basis of the mean value curve of the instantaneous frequency of the modal functions (IVMD), subsequently fusing these multi-scale modal components with a DCNN to automatically learn fault characteristics and build a bearing fault diagnosis model [19]. Jiang et al. proposed a coarse-to-fine decomposition strategy, initially setting balance parameters roughly and then finely tuning them to identify the optimal target modes [20]. These analyses, traditionally focusing on optimizing a single parameter, highlight the need for an algorithm capable of simultaneously optimizing two parameters. Such an approach would improve the adaptability, global search ability, and computational efficiency of VMD, allowing for more effective extraction of fault features from signals.

Recent advancements in the field of bearing fault diagnosis have effectively leveraged Variational Mode Decomposition (VMD) alongside advanced fault feature extraction and pattern recognition technologies, achieving notable results. For instance, Jiang et al. have effectively identified rolling bearing faults by combining VMD with fuzzy c-means clustering techniques [21]. Zhang et al. employed the Improved Particle Swarm Optimization (IPSO) to refine the selection of optimal modal components and penalty factors for decomposing vibration signals, applying it to the decomposition of vibration signals [22]. These approaches typically rely on traditional “shallow learning” pattern recognition algorithms and require manual feature extraction post-signal decomposition. While effective, their expressive power and generalization capabilities exhibit certain limitations when addressing complex functions.

In recent years, researchers have employed various deep learning models to address bearing fault signals. Unlike traditional diagnostic methods that depend on manual intervention and expert knowledge, deep learning leverages deep networks to automatically extract and classify features through successive layers of nonlinear activations, fundamentally reducing reliance on human expertise. Chen et al. introduced a multi-scale convolutional neural network and long short-term memory (MCNN-LSTM) fault diagnosis model, which incorporates dual CNNs with varying kernel sizes to enhance feature extraction capabilities prior to classification through an LSTM network [23]. Mao et al. developed a semi-stochastic subspace approach that employs bidirectional gated recurrent units (Bi-GRU), effectively blending statistical techniques with deep learning for enhanced information extraction [24]. Zhao et al. proposed a convolutional gated recurrent unit (ConvGRU) relational network for fault diagnosis, which generates class prototypes using ConvGRU and measures their similarity to query set features through a relational module to calculate relationship scores, thereby achieving fault diagnosis [25]. However, these methods often overlook the long-term dependencies hidden within time-series data, potentially leading to information loss.

This article introduces an integrated composite deep learning architecture that combines CPO-VMD with a convolutional neural network, equipped with bidirectional gated recurrent units and a self-attention mechanism. Initially, the CPO algorithm optimizes VMD parameters to achieve more effective signal decomposition. Subsequently, VMD decomposes vibration and acoustic signals into multiple intrinsic mode functions, which are then processed through the CNN-BiGRU-AT network to capture temporal dependencies within the signals. These undergo detailed feature extraction across multiple stages of convolution and pooling, manifesting their time-frequency attributes. The bidirectional gated recurrent units, with their acute capture of temporal features, incorporate long-term dependencies and both forward and reverse signal flows, enriching the feature representation. The crucial self-attention layer then activates, prioritizing and highlighting critical sequential segments before feeding the extracted vibro-acoustic features into a fully connected layer to facilitate the feature fusion process. The output layer classifies the features, completing the task of bearing fault diagnosis. Experimental validation of this method indicates it achieves high accuracy and robust performance in identifying bearing faults. The following highlights our contributions.

(1) The proposed method directly processes fused acoustic-vibro signals without the need for manual feature extraction, leveraging the strengths of VMD and CPO for optimized signal decomposition.
(2) The CNN-BiGRU-AT network effectively extracts both spatial and temporal features from the decomposed intrinsic mode functions (IMFs), resulting in more accurate bearing fault diagnosis.
(3) The BiGRU layers in the CNN-BiGRU-AT architecture follow the convolutional layers, significantly reducing the number of time steps and thus the computational complexity associated with processing long sequences.
(4) The proposed solution demonstrates strong adaptability and robustness across varying working conditions and signal-to-noise ratios, making it highly suitable for real-world industrial applications.

## 2 Basic principles

A. Variation mode decomposition theory

Variational Mode Decomposition (VMD), a technique proposed by Dragomiretskiy and Zosso, represents a cutting-edge advancement in signal processing [15]. This method elegantly decomposes a complex signal into an ensemble of proposed orthogonal intrinsic modal functions (IMFs) through a non-recursive approach. Typically, in the VMD process, a signal f(t) is methodically separated into multiple modes $u_{k}\left(t\right)$, as delineated in equation (1). This approach allows for a precise and structured analysis of signals, serving as a robust tool in the signal processing domain.

(1) $$f\left(t\right)=\sum_{k=1}^{N}u_{k}\left(t\right)$$

where N is the number of modes in the decomposition, $u_{k}\left(t\right)$ is a narrowband mode, which can be described essentially as an amplitude modulation-frequency modulation (AM-FM) signal, defined as

(2) $$u_{k}\left(t\right)=A_{k}\left(t\right)\times \cos\left(\phi_{k}\left(t\right)\right)$$

where $A_{k}\left(t\right)$ is an instantaneous assignment of $u_{k}\left(t\right)$ and $\phi_{k}\left(t\right)$ is a non-decreasing phase function.

Utilizing the Hilbert transform, the one-sided spectrum of each analyzed signal is computed. This is followed by a precise adjustment of the center frequencies predicted by each modal function, ensuring the modulation of the spectrum to the corresponding fundamental frequency band. A refined measure of the spread is obtained by determining the bandwidth of each Intrinsic Modal Function (IMF) based on the bound norm L2. VMD is elegantly constrained by the objective of minimizing the total bandwidths across all modes, culminating in a constrained variational model, which is articulated as follows:

(3) $$\left\{\begin{array}{c} \begin{array}{c} \min\\ \left[uk,\omega k\right] \end{array}\left\{\sum_{k}\|\partial_{t}[\left(\delta \left(t\right)+\frac{j}{\pi t}\right)*u_{k}\left(t\right)]e^{-j\omega kt}\|\begin{array}{c} 2\\ 2 \end{array}\right\}\\ s.t.\sum_{k=1}^{K}u_{k}=f\left(t\right) \end{array}\right\}$$

where K represents the number of IMFs, $u_{k}$ denotes the kth IMF, $\omega_{k}$ is the center frequency of the kth IMF, and δ(t) is the Dirac function.

B. Crested Porcupine Optimizer (CPO)
1. principle

The Crested Porcupine optimization algorithm (CPO) [26] is a natural meta-heuristic algorithm proposed by Abdel-Basset et al., in 2024, inspired by the defense strategies of Crested Porcupines (CPs). The crested porcupine, a large rodent, typically resides in small family groups, yet it often relies on its formidable defense strategies when foraging alone. These creatures possess a repertoire of unique defensive tactics that they can adapt flexibly based on the nature and urgency of the threat, exhibiting a multi-layered defense strategy ranging from vigilance to aggression. This adaptability enables them to effectively counter potential predators and competitors. Their defensive behaviors can be categorized into four primary modes, each escalating in aggressiveness, thus affording them robust protection and the capacity to thrive in their environments.

**Fig. 1:**
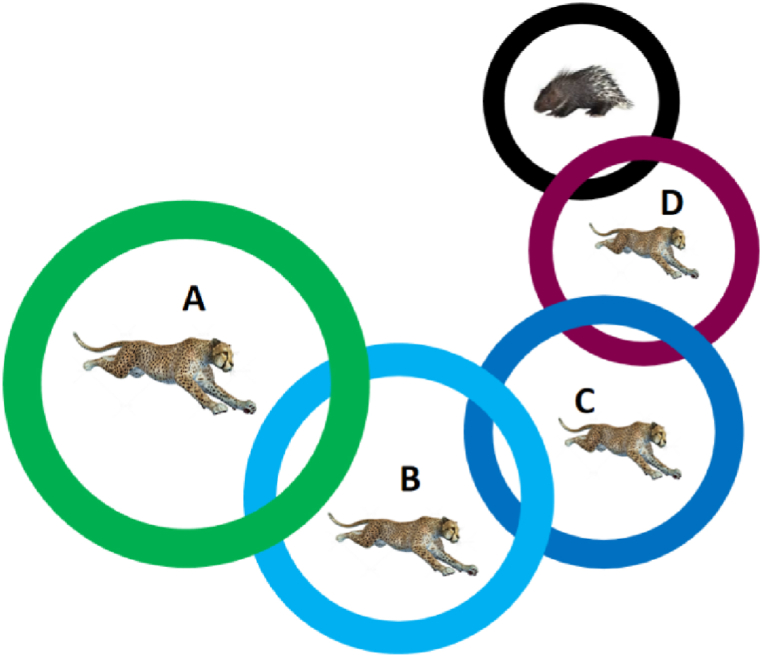
Defense policy area: A indicates that the first defense mechanism will be activated; B indicates the second defense mechanism; C indicates that the third defense mechanism; D indicates that the fourth defense mechanism.

**Fig. 2:**
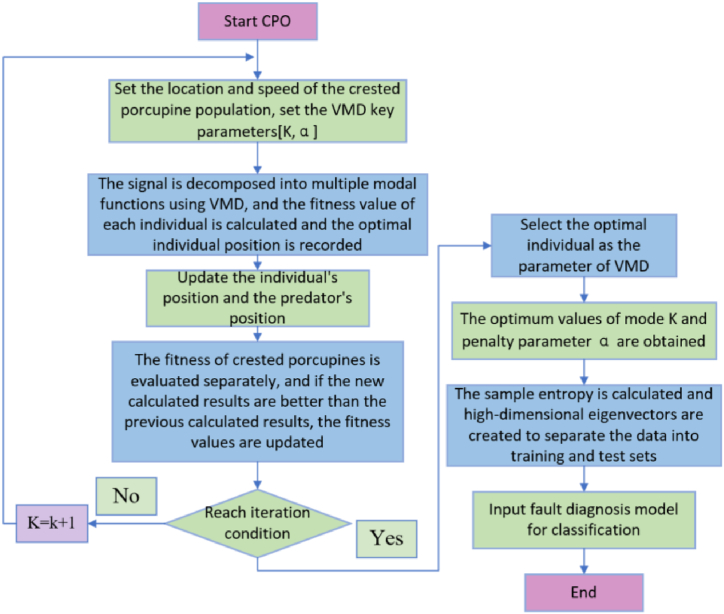
Flowchart of CPO-VMD.

The CPO algorithm mimics the crested porcupine's multi-layered defense strategy, ranking it in order of its aggressiveness: sight, sound, smell, and aggression. In CPO, the search space is divided into four regions, each corresponding to a defense behavior, as shown in Fig. 1. The first zone (A) represents the initial defense zone, where the crested porcupine, keeping a safe distance from predators, employs its first line of defense. This zone corresponds to the use of visual detection to identify potential threats early on. The second zone (B) comes into play if the predators are undeterred by the initial measures and continue their approach. In this phase, the porcupine utilizes auditory signals as a secondary defensive mechanism, akin to a warning or deterrent. The third zone (C) is activated when the predators show resilience to the previous strategies and draw even closer. Here, the porcupine relies on its keen sense of smell to further assess the situation, representing a more focused and deliberate defensive posture. Finally, the last zone (D) signifies the ultimate defense phase, where, after the failure of all prior defenses, the porcupine resorts to direct physical aggression. In this critical zone, the porcupine launches an aggressive attack to incapacitate or even kill the predators, ensuring its survival. This multi-tiered defense strategy reflects the porcupine's adaptive and escalating response to threats, progressively intensifying its defenses as the danger becomes more imminent.In the algorithm, visual and sound defense strategies are used to explore a wide range of possible solutions, while odor and attack strategies are used to exploit local optimal solutions more precisely. Through this combination of exploration and exploitation, CPO algorithms aim to effectively optimize problem-solving strategies that mimic the survival strategies of crested porcupines in nature (see Fig. 2).

2. Mathematical model

According to the defense behavior of CPs, when predators are far away, CPs have two defense strategies, namely, visual strategy and sound strategy. These two strategies are mainly for exploration and search, so that CPs can scare predators away without attacking them. When predators are close to CPs, CPs turns to odor and attack strategies and makes use of these defense strategies. Try to simulate the reaction of predators away from CPs after defensive behavior to explore the safest area, and then approach or reach the optimal solution.

When CPs sense the presence of a predator, they flapping their feathers to create the illusion that it is larger, thereby influencing the predator's choice of behavior: approach or move away. If a predator chooses to approach, the distance between the two is shortened, which helps accelerate exploration and convergence of the neighboring area. Conversely, if the predator chooses to stay away, it will increase its distance from the CPs, a strategy that helps to explore further unknown areas for possible solutions. The normal distribution is used to generate random values to mathematically model these decision processes:

(4) $$\vec{x_{i}^{t+1}}=\vec{x_{i}^{t}}+\tau_{1}\times \left|2\times \tau_{2}\times \vec{x_{CP}^{t}}-\vec{y_{i}^{t}}\right|$$

Where $\vec{x_{CP}^{t}}$ is the optimal value of the function at the *t* iteration; $\vec{y_{i}^{t}}$ Is the vector generated between the current CP and a randomly selected CP in the population to represent the position of the predator at the *t* iteration; $\tau_{1}$ is a random number based on a normal distribution; $\tau_{2}$ is a random value in the interval [0,1]. $\vec{y_{i}^{t}}$ is as follows:

(5) $$\vec{y_{i}^{t}}=\frac{\vec{x_{i}^{t}}+\vec{x_{r}^{t}}}{2}$$

Where *r* is the random number between [1,N].

In the second defense strategy, CPs make noise and threaten the predator. When the predator approaches, CPs will increase its volume, but when the sound is weak, the predator will continue to move toward CPs, when the sound is louder, the predator will stay in place, and when the noise is loud, the predator will move away from CPs because of fear. To simulate this behavior mathematically, the following formula is proposed:

(6) $$\vec{x_{i}^{t+1}}=\left(1-\vec{P_{1}}\right)\times \vec{x_{i}^{t}}+\vec{P_{1}}\times \left(\vec{y}+\tau_{3}\times \left(\vec{x_{r_{1}}^{i}}-\vec{x_{r_{2}}^{i}}\right)\right)$$

Where $r_{1}$ and $r_{2}$ are two random integers between [1,N]; $\tau_{3}$ is a random value generated between 0 and 1; $\vec{P_{1}}$ is a random binary vector containing 0 and 1 to prevent predators from treating the noise response in the same way. When the unit in the vector contains 0, it means that the predator will stay in place. When the cell in the vector contains 1, it means that the predator may continue to move toward or away from the CPs.

In the third defense strategy, CP secretes a foul odor to spread in the surrounding area in order to prevent predators from approaching. In order to simulate this behavior mathematically, the following formula is proposed:

$$\vec{x_{i}^{t+1}}=\left(1-\vec{P_{1}}\right)\times \vec{x_{i}^{t}}+\vec{P_{1}}\times$$

(7) $$\left(\vec{x_{r_{1}}^{i}}+S_{i}^{t}\times \left(\vec{x_{r_{2}}^{i}}-\vec{x_{r_{3}}^{i}}\right)-\tau_{3}\times \vec{\delta }\times \gamma_{t}\times S_{i}^{t}\right)$$

Where $r_{3}$ a random number between [1, N); δ is to control the parameters of the search direction; $\vec{x_{i}^{t}}$ is the position of the *i*th individual in the *t* iteration; $\gamma_{t}$ is the defense factor; $\tau_{3}$ is the random number between the interval [0,1]; $S_{i}^{t}$ Is the odor diffusion factor. The following formula of δ、 $\gamma_{t}$ and $S_{i}^{t}$ were proposed:

(8) $$\vec{\delta }=\left\{ \begin{array}{c} +1,if\;\vec{rand}\leq 0.5\\ -1,Else \end{array}\right.$$

(9) $$\gamma_{t}=2\times rand\times {\left(1-\frac{t}{t_{\max}}\right)}^{\frac{t}{t_{\max}}}$$

(10) $$S_{i}^{t}=\exp\left(\frac{f\left(x_{i}^{t}\right)}{\sum_{k=1}^{N}f\left(x_{i}^{t}\right)+\epsilon }\right)$$

Where $f\left(x_{i}^{t}\right)$ represents the value of the objective function of the ith individual at iteration t; At iteration t, ϵ is a smaller value to avoid dividing by zero; $\vec{rand\;}$ is a random vector of values generated between 0 and 1; *rand* is a randomly generated numerical variable between 0 and 1; *N* is the population size; *t* is the number of current iterations and $t_{\max}$ is the number of iterations.

The strategy simulates three possible situations with the vector $\vec{P_{1}}$: (1) when $\vec{P_{1}}$ = 0, the predator stops moving due to fear of CP, CP will stop odor diffusion, so the distance between the predator and CP remains unchanged; (2) when $\vec{P_{1}}$ = 1, CP emits a distinct odor due to the presence of predators; (3) when $\vec{P_{1}}$ is a combination of 0 and 1, a certain safe distance is maintained between the predator and CP, and a large amount of odor release is not required.

The fourth defense strategy is physical attack. When a predator is very close, CP will adopt a physical attack, during which the two objects come into close contact, manifesting as a one-dimensional inelastic collision. In order to express this physical attack behavior in a mathematical formula, the following formula is proposed:

$$\vec{x_{i}^{t+1}}=\vec{x_{CP}^{t+1}}+\left(\alpha \left(1-\tau_{4}\right)+\tau_{4}\right)\times$$

(11) $$\left(\delta \times \vec{x_{CP}^{t+1}}-\vec{x_{i}^{t}}\right)-\tau_{5}\times \delta \times \gamma_{1}\times \vec{F_{i}^{t}}$$

Where α Is the convergence rate factor; *τ*4 is the random value generated in the interval 0,1; *Fti* represents the average force generated by the crested porcupine against the *i* predator. The definition of this force follows the inelastic collision law and is defined by equation (12) as follows:

(12) $$\left\{ \begin{array}{c} \vec{F_{i}^{t}}=\vec{\tau_{6}}\times \frac{m_{i}\times \left(\vec{v_{i}^{t+1}}-\vec{v_{i}^{t}}\right)}{\Delta t}\\ m_{i}=\frac{f\left(\vec{x_{i}^{t}}\right)}{e^{\sum_{k=1}^{N}f\left(\vec{x_{k}^{t}}\right)+\epsilon }}\\ \vec{v_{i}^{t}}=\vec{x_{i}^{t}}\\ \vec{v_{i}^{t+i}}=\vec{x_{r}^{t}} \end{array}\right.$$

Where $m_{t}^{i}$ is the mass of the i th individual at the t th iteration; f(∙) represents the objective function; $\vec{v_{i}^{t+1}}$ is the final velocity of the i th individual at the next iteration *t*+1, assigned based on randomly selected solutions from the current population; $\vec{v_{i}^{t}}$ is the initial velocity of the *i* th individual when iterating t; Δt is the number of iterations; $\vec{\tau_{6}}$ is a vector containing random values generated between [0,1].

In equation (12), the mean force $F_{t}^{i}$ of CP is calculated by dividing the molecule by the current number of iterations, and it increases linearly during optimization, so that the influence of the mean force of CP gradually decreases. In practice, a smaller value of this factor is detrimental to the performance of CPOs because they may not be able to use the various regions around the optimal solution to find a better solution. Therefore, this equation can be updated by removing the numerator and relying only on the denominator, as shown in equation (13). This approach helps to create a broad set of values within the search space, allowing for a more comprehensive examination of the area around the best solution. The advantage of this approach is that it accelerates the convergence to the approximately optimal solution by focusing development into the area around the optimal solution.

(13) $$\vec{F_{i}^{t}}=\vec{\tau_{6}}{\times m}_{i}\times \left(\vec{v_{i}^{t+1}}-\vec{v_{i}^{t}}\right)$$

C. VMD based on CPO improvement

VMD is an adaptive method for signal decomposition that optimizes performance by tuning key parameters such as the penalty factor α and the number of modes *K*. During the initialization of VMD parameters, it is essential to set these two parameters. Additionally, while convergence error ε and fidelity coefficients γ are typically initialized, empirical evidence suggests that their impact on outcomes is negligible, thus default values are often employed. The selection of the modal count *K* and the penalty factor α is crucial; inappropriate choices can lead to either over-decomposition or incomplete decomposition, and an unsuitable penalty factor may affect the decomposition speed and the bandwidth of the components.

This paper implements a global search on VMD using CPO, establishing an CPO-VMD parameter optimization model.

In the optimization of VMD parameters, the initial step requires the selection of a fitness function for the CPO algorithm. For this paper, sample entropy has been selected as the fitness function, with its calculation defined by the following formula:

(14) $$SampEn=-\ln\left(\frac{A_{M+1}\left(r\right)}{B_{M}\left(r\right)}\right)$$

Where *M* is the sequence length and *r* is the threshold value.

The specific implementation is as follows.

1. Acquire vibro-acoustic data signals from rolling bearings under various fault conditions and in healthy states.
2. Initialize algorithm parameters including population size *N* and maximum number of iterations *Iter*_*max.*_ The raw signal is decomposed into multiple modal functions using VMD.
3. In each iteration, the fitness of each individual in the population is assessed according to the objective function of the VMD. Use the CPO position update formula to adjust the VMD parameters, that is, to update the CP position. This process involves updating the position of the remaining individuals and updating the position of the predator.
4. Compute the updated position information for those individuals who perceive danger, calculate the fitness of the updated CP individuals, and update if the new fitness value is better than the former one.
5. Based on the optimized parameters, perform VMD signal decomposition to obtain each mode's signal component.
6. Compute the sample entropy for each IMF component, select the optimal IMF component as the feature vector, and partition this vector into training and testing sets for input into the fault classification model.
D. CNN-BiGRU-AT based on acoustic vibration fusion

A fault diagnosis method of CNN-BiGRU-AT based on acoustic-vibration fusion algorithm is proposed for bearing fault diagnosis. In this method, the feature vector obtained from CPO-VMD decomposition of acoustic and vibration original signals is input as the fusion data set, and then the signals are analyzed by intelligent classification technology to accurately identify the bearing fault.

**Fig. 3:**
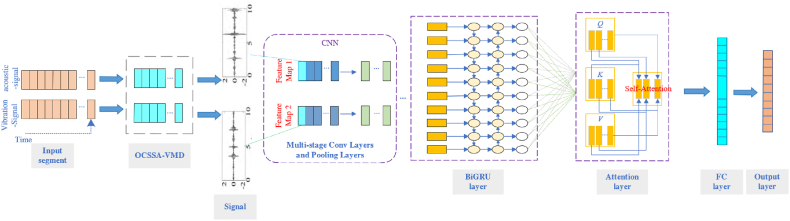
CNN-BiGRU-AT algorithm based on acoustic-vibration fusion.

This enhancement significantly increases the precision of fault detection in bearings. Specifically, the algorithm operates through three distinct phases: extracting features, fusing features, and classifying faults. Fig. 3 shows the.

Algorithm framework. The multi-modal sensor feature extraction phase of the algorithm involves extracting features from both vibration and acoustic signals. Initially, the algorithm utilizes the CPO-VMD algorithm to decompose the original time-series signals, which are then processed through a CNN for further feature extraction. The CNN consists of four convolutional layers and four pooling layers, each applied sequentially to acoustic-vibration signals. These operations are repeated four times, with the specific parameters detailed in Table 1. The resulting feature maps undergo subsampling through the pooling layers, a process that not only reduces the dimensions of features but also preserves essential information, thus enabling the network to withstand minor variations in input data. As the data flows through the alternating convolutional and pooling layers, the model autonomously learns and extracts nonlinear features embedded within the raw data. After completing the feature extraction process, the output from the final pooling layer is fed into the BiGRU network. The BiGRU is specifically engineered to capture the temporal dynamics of time-series data, including long-term dependencies and both forward and backward information flows, thus offering a comprehensive view of temporal dynamics. Subsequently, the attention mechanism further enhances the model’s capability to process temporal features by assigning varying weights to each element in the sequence, thereby highlighting important features and enabling the model to intelligently prioritize critical information. The output from the BiGRU-AT is channeled into a fully connected layer, which integrates and transforms features for classification. This layer serves to amalgamate all extracted vibrational and acoustic features into a coherent whole, facilitating the feature fusion process. Table 2 shows the fusion layer configuration. Ultimately, the output layer is tasked with mapping these fused features onto fault categories, finalizing the diagnostic process.

**Table 1:** Model parameter.

No.	Layer type	Kernel size	Kernel channel size
1	Convolution 1	64 x 1	16
2	Pooling 1	16 x 1	16
3	Convolution 2	32 x 1	64
4	Pooling 2	2 x 1	64
5	Convolution 3	32 x 1	128
6	Pooling 3	2 x 1	128
7	Convolution 4	16 x 1	128
8	Pooling 4	2 x 1	128

**Table 2:** Model parameter.

No.	Layer type	Units
1	Fully-connected	120

**Fig. 4:**
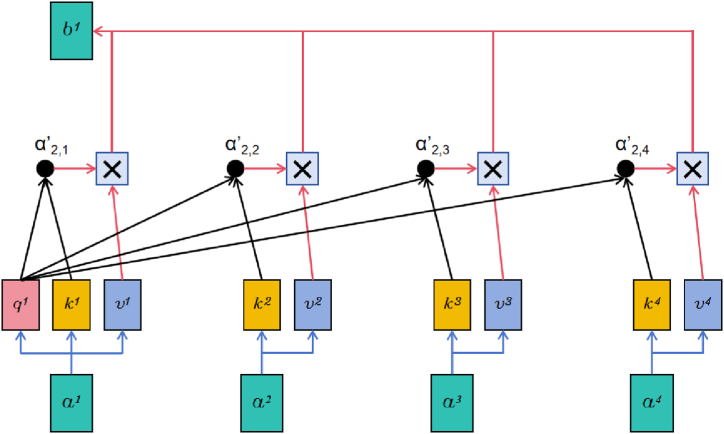
Core idea of attention mechanism.

In the BiGRU layer, an enhanced focus on pivotal temporal features is achieved through the integration of a self-attention mechanism. The essence of this attention mechanism lies in assigning higher weights to critical information within the data while disregarding irrelevant details. This enables the model to delve deeper into and leverage key features more effectively. Particularly during the operation of bearings, where vibrational characteristics may vary with different operational conditions, the dynamic adjustment of focus points by the self-attention mechanism allows the model to adapt flexibly to these changes, thereby maintaining a high level of diagnostic performance stability. Fig. 4 show the core idea of self-attention mechanism.

In Fig. 4, $a^{i}$ is multiplied by weight matrices $W^{q}$, $W^{k}$ and $W^{v}$ to yield matrices $q^{i}$, $k^{i}$ and $v^{i}$ respectively. $q^{i}$ assesses the interaction level of the current element with other elements in the sequence, $k^{i}$ indicates the correlation with $a^{i}$ and the query, $v^{i}$ represents the critical information of $a^{i}$. $a_{i.j}^{\prime }$ is obtained by normalizing the computation of $q^{i}$ and $k^{i}$ to represent the similarity between the elements, which is then multiplied with $v^{i}$ and accumulated to get the output $b^{i}$. The computation formula, as depicted in Equation (8), considers information from all positions within the sequence. Thus, the attention mechanism adaptively captures long-range dependencies between elements, enhancing the model’s ability to dynamically understand complex relationships within the data.

(15) $$b^{i}=\sum_{i=1}^{N}a_{i.j}^{\prime }v^{i}$$

## 3 Experiments

E. Experimental platform and data collection

**Fig. 5:**
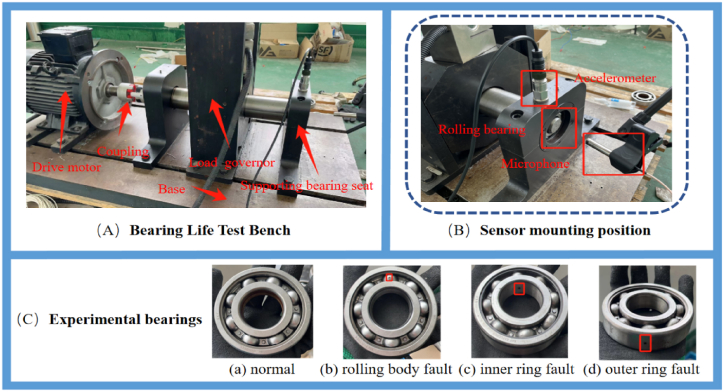
Bearing life test rig: （A） experimental setup（B） sensor mounting positon（C）Experimental bearings.

In this experiment, bearing acoustic and vibration data were collected using the bearing life test rig as illustrated in Fig. 5. This system comprises a load-bearing seat, a motor drive unit, a load device, a signal acquisition device, and a supervisory computer. To validate the performance of the proposed algorithm, accelerometers and microphones were employed to gather acoustic and vibration data from 10 experimental groups. The bearings utilized were SKF 6308 deep groove ball bearings, the specifics of which are detailed in Table 3. To simulate bearing fracture failures, holes were introduced into the inner and outer rings and the roller using electric spark machining, as shown in Fig. 5(C). The experimental samples included faults with hole diameters of 1 mm, 1.5 mm, and 2 mm on the inner and outer rings and rollers, plus normal bearings, totaling 10 types of samples. The motor speed was set at 1500 rpm. The entire signal acquisition setup included a data acquisition card and single-channel vibration and acoustic sensors. The data acquisition card used was the NI 9231, which allows for high dynamic range measurements to fully utilize modern measurement microphones and accelerometers. The collection and preliminary processing of the experimental data were executed using LabVIEW programming. The sampling rate was set at 4.8 kHz, and a dataset comprising vibration and acoustic signals was collected from ten different sets of bearings. Each set randomly collected 2000 samples, each containing 4800 vibration signal points and 4800 acoustic signal points.

F. parameter optimization

**Table 3:** Parameters of bearing used in the experiments.

Bearing type	Pitch Diameter	Roller Diameter	Roller Number
SKF 6308	90（mm）	40（mm）	8

Fig. 7 illustrates the time-domain waveforms of vibration and acoustic signals collected from ten groups of bearings. The waveforms reveal distinct shock components and noise interference within these signals, while their spectral contents remain rich. Notably, the type of fault cannot be determined solely based on these waveforms; however, it is intriguing that the vibration and acoustic signals exhibit consistent patterns at the moment of shock when under the same fault condition. The acoustic-vibration signals were processed using the methodology described in this paper, with a predefined modal number *K* = 6 and a penalty factor α of 2500. For the CPO optimization, the population size *n* was set at 20, and the maximum number of iterations *L* was limited to 15. Table 4 presents the optimally derived parameter combinations *K* and α for the ten fault modes as identified by the CPO (see Fig. 8).

**Fig. 6:**
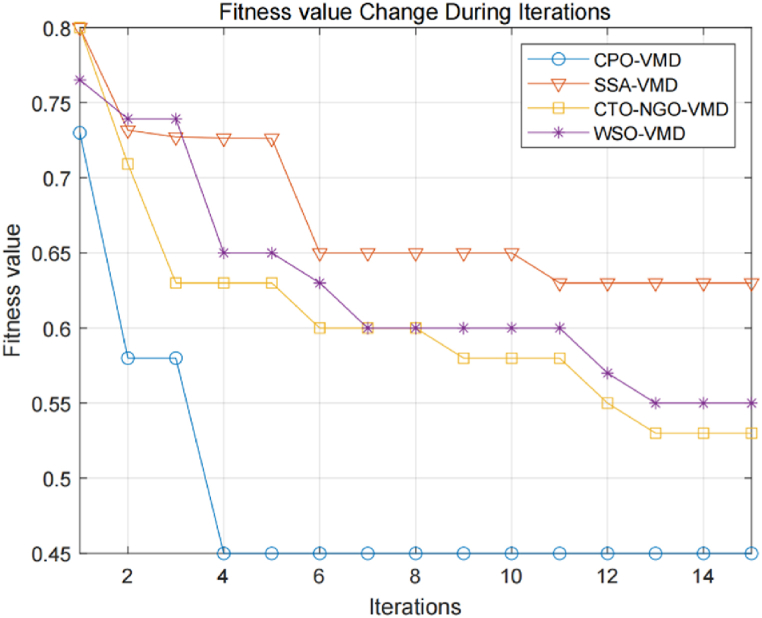
The change curve of fitness value.

**Fig. 7:**
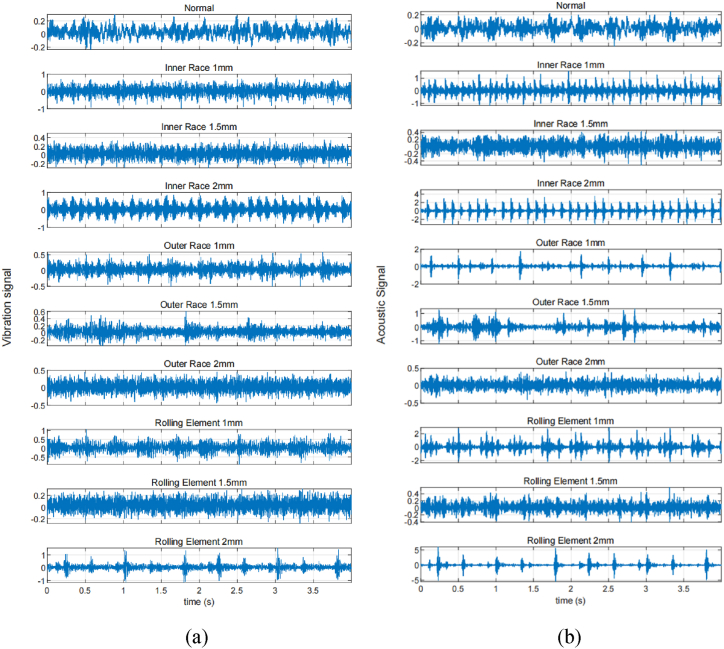
Original waveforms of bearings in ten failure modes (a) vibration signal (b) acoustic signal.

**Fig. 8:**
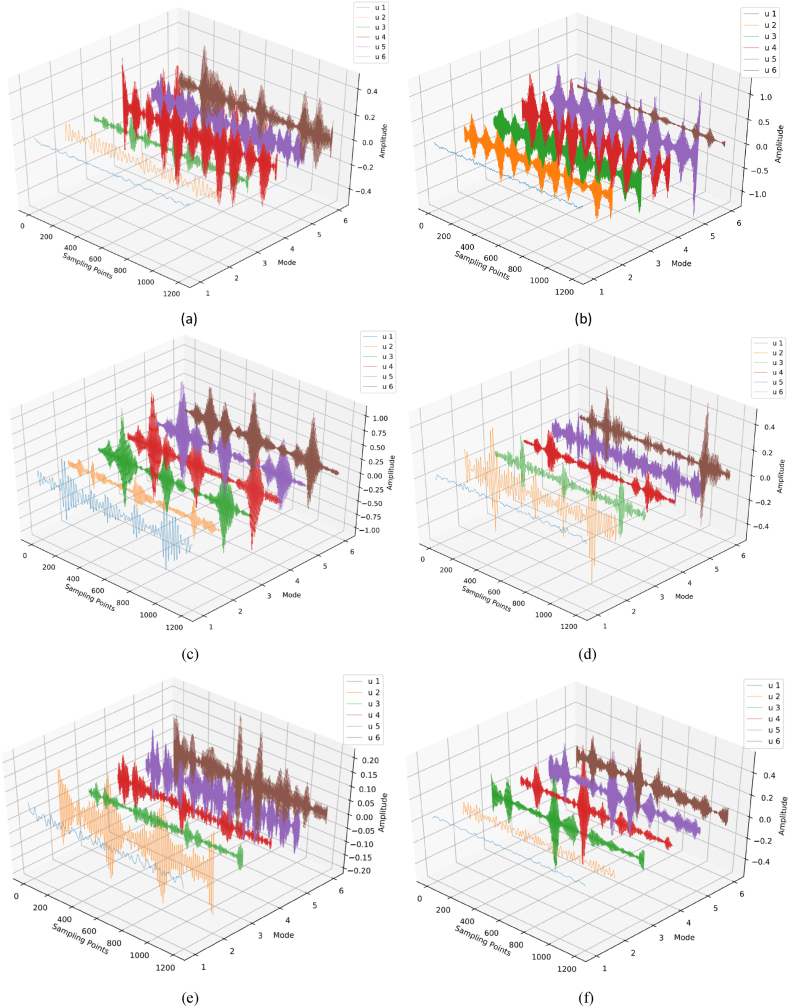
CPO optimal parameter decomposition of fault signals (a) Vibration signal outer ring (b) acoustic signal outer ring (c) Vibration signal inner ring (d) acoustic signal inner ring (e) Vibration signal rolling body (f) acoustic signal rolling body.

**Table 4:** Optimal penalty factors and modal components of acoustic-vibration signals.

Fault Mode	Vibration Signal		Acoustic Signal	
	α	*K*	α	*K*
**Normal**	110	10	1316	6
**Inner 1 mm**	2104	10	1812	10
**Inner 1.5 mm**	1109	6	1887	6
**Inner 2 mm**	2427	9	1515	8
**Outer 1 mm**	2500	5	2469	6
**Outer 1.5 mm**	421	6	2336	5
**Outer 2 mm**	2372	10	2405	10
**Roller 1 mm**	2280	6	1188	6
**Roller 1.5 mm**	2271	10	869	10
**Roller 2 mm**	2491	10	2292	10

## 4 Ablation analysis of the proposed method

Experiment 1: Comparative Analysis of CPO Optimization Algorithm:

To demonstrate the superiority of the CPO algorithm in optimizing VMD, a comparative analysis was conducted against SSA, CTO-NGO, and WSO, using optimization time and decomposition time as evaluation metrics. The calculated results, as shown in Table 5, highlight the respective optimization and decomposition times for each method. The change curve of the fitness value during the iterative update of each algorithm is depicted in Fig. 6. This comparison underscores the enhanced capability of the CPO-VMD approach in efficiently isolating relevant features from complex signals. It is important to note that sample entropy was used as the fitness function for all the methods to ensure comparability of the results.

**Table 5:** Algorithm parameters and processing time.

algorithm	parameters	value	Optimization time/s	VMD decomposition time/s
CPO	Step Size	[0.5,1]	102.55	0.62
	Radius for Defense Zone 2	[0,1]		
	Maximum Number of Iterations	15		
WSO	Random Parameter	[0,1]	121.88	0.68
	Convergence Rate Parameter	0.5		
	Number of Agents	10		
SSA	Step Size	[0.5,1]	924.21	0.82
	Swarm Range Parameter	[0,1]		
	Maximum Number of Iterations	15		
CTO-NGO	Radius for Cooperation Zone 1	[0,1]	141.92	0.98
	Radius for Cooperation Zone 2	[0,2]		
	Number of Cooperating Groups	1 or 2		

From Fig. 6, it can be seen that the CTO-NGO-VMD algorithm achieves a faster convergence time, completing the optimization in fewer iterations; however, this comes at the cost of compromised accuracy, as indicated by its higher final fitness value compared to other methods. Conversely, the WSO-VMD algorithm excels in convergence accuracy, reaching the lowest fitness value, but it converges at a slower rate, requiring more iterations to achieve optimal results. The SSA-VMD algorithm, meanwhile, exhibits neither high convergence speed nor accuracy, as it takes a longer time to converge and results in a higher final fitness value, rendering its performance suboptimal. In contrast, the CPO-VMD algorithm ensures high convergence accuracy with rapid rates, achieving a fitness value close to the optimal one in just a few iterations, thus outperforming the other three algorithms in overall efficacy. Table 5 further illustrates that the CPO-VMD method presented in this study optimizes the VMD algorithm more efficiently than alternative approaches, with a significantly lower optimization time of 102.55 s and VMD decomposition time of 0.62 s, demonstrating a balance between convergence speed and accu racy.

**Fig. 9:**
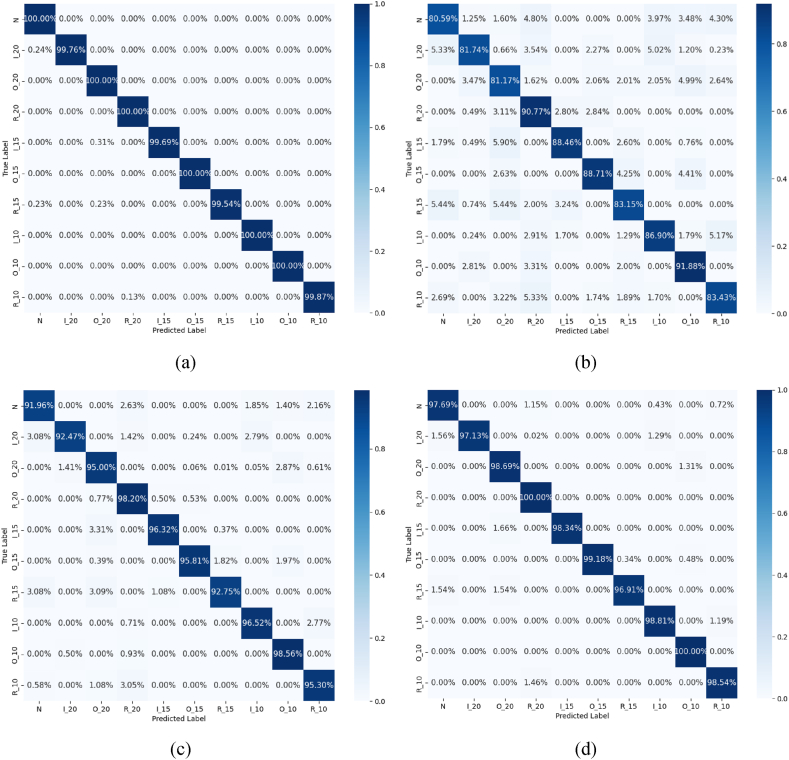
Confusion matrices representing the fault classification results following VMD optimization via (a) CPO; (b) SSA; (c) WSO; (d) COT-NGO.

Subsequently, this study will verify the effectiveness of the CPO-VMD, which is selected as the standard classification model for this study based on the solid theoretical foundation and extensive practical application experience established within the field of bearing fault diagnosis by the VMD-CNN-BiLSTM method. In the study, the raw data of vibration signals are optimized by VMD decomposition by CPO, SSA, WSO, and CTO-NGO methods, and the obtained IMF components are input into CNN-BiLSTM-AT for classification. As shown in Fig. 9, the accuracy of fault detection is 99.89 %, 85.68 %, 95.29 % and 98.53 %, respectively, and these data fully demonstrate the effectiveness of the CPO-VMD decomposition strategy. The diagnostic accuracy of SSA is the lowest because SSA tends to fall into local optima, especially in complex optimization problems. Due to the relatively weak global search capability of SSA, it may fail to effectively find the global optimum in multimodal signal processing. As a result, the VMD decomposition optimized by SSA may not accurately extract the most discriminative signal features, thereby affecting classification accuracy. The WSO algorithm, while possessing a degree of global search capability, exhibits slower convergence, particularly in the context of complex signal processing tasks. This often necessitates a greater number of iterations to achieve an optimal solution, making WSO less effective under constrained time and resource conditions compared to other algorithms. In contrast, the CTO-NGO algorithm, a hybrid approach combining bee colony and vortex search strategies, demonstrates robust global search capabilities while simultaneously excelling at local feature extraction.

Experiment 2: Comparison of each algorithm in unimodal and multimodal experiments.

The efficacy of the CPO-based VMD for modal decomposition of acoustic vibration signals has been thoroughly analyzed and verified, establishing a robust foundation for further exploration of its applications in bearing fault diagnosis. The forthcoming study will delve into the effectiveness of an acoustic-vibration feature fusion method. This method involves feeding feature vectors derived from CPO-VMD decomposition into an acoustic-vibration fusion-based CNN-BiGRU-AT model to construct a diagnostic system that integrates acoustic and vibration features. The performance of this system will be evaluated under three distinct scenarios: vibration signal, acoustic signal, and acoustic-vibration signal fusion. By conducting a comparative analysis with classical algorithms such as CNN-BiLSTM, 1D-CNN, WDCNN, and CNN-BiGRU, the study seeks to elucidate the efficiency and accuracy of various signal decomposition and feature extraction methodologies in identifying bearing faults, thereby highlighting the potential benefits of integrating CPO-VMD with the multimodal CNN-BiGRU-AT model in this domain.

**Fig. 10:**
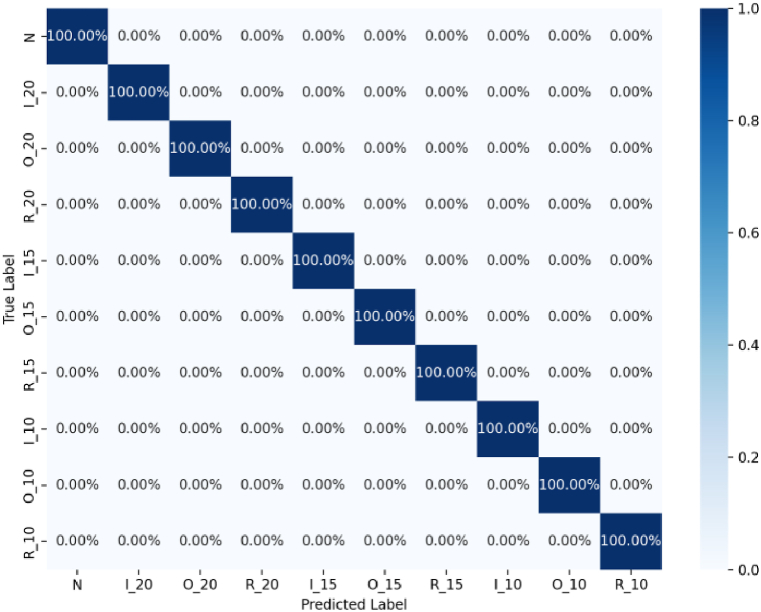
The confusion matrix for the methods proposed in this study.

Sample entropy is computed for each IMF component to identify the component with the lowest sample entropy, which is then selected as the feature vector. Seventy percent of each is allocated as the training set and the remaining thirty percent as the test set. These sets are inputted into the CNN-BiGRU-AT network model, designed to diagnose faults by fusing acoustic and vibration data. The model reached a accuracy of 100 % in testing, and its confusion matrix is depicted in Fig. 10, further validating the rationality and effectiveness of the proposed method.

**Fig. 11:**
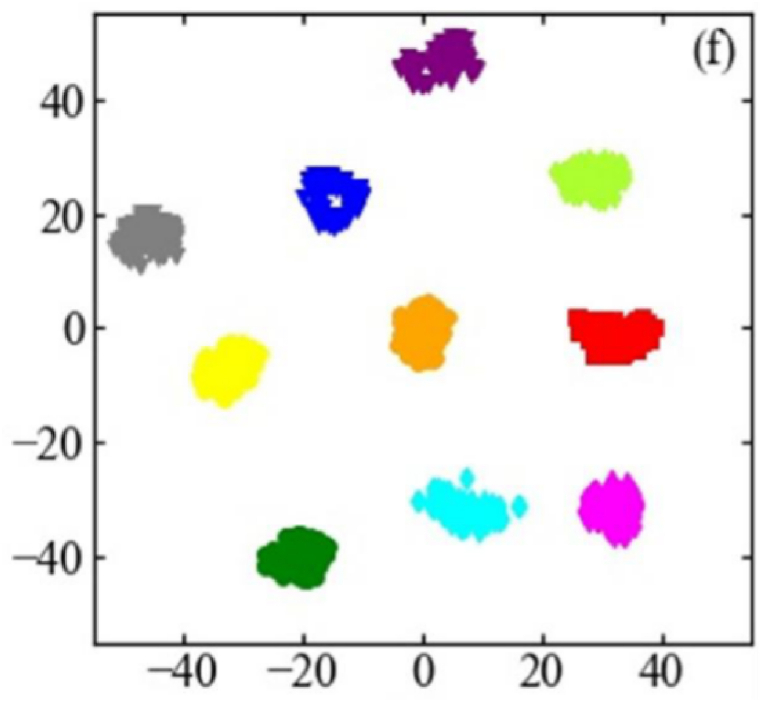
t-SNE feature visualization results.

In order to elucidate the feature learning capabilities of the proposed method, t-SNE is employed to visualize the features learned at each layer, with the outcomes displayed in Fig. 11. Following deep feature extraction from the fused output of acoustic vibration signals, points corresponding to the same category are clustered together, while those from different categories are distinctly separated. This spatial arrangement enables effective fault classification using the features derived from this layer.

**Fig. 12:**
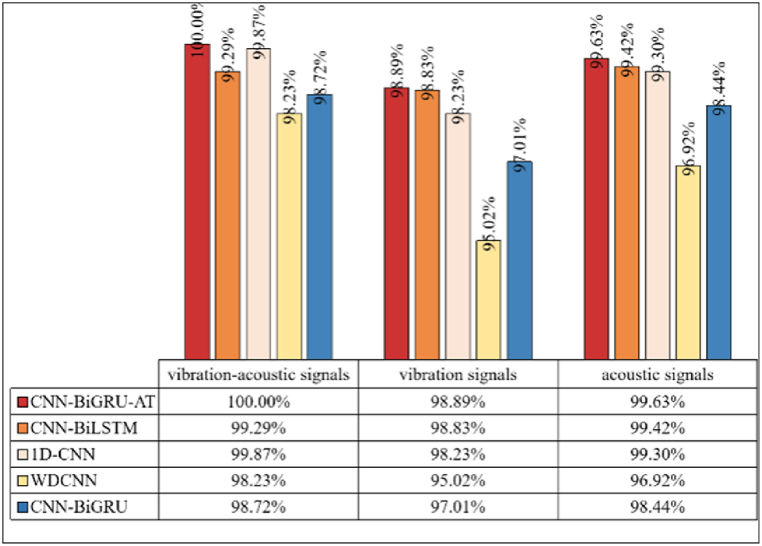
Accuracy of different models.

The data types utilized by various methods and their diagnostic outcomes are illustrated in Fig. 12. The method introduced in this study leverages the full potential of acoustic-vibrational signal fusion, achieving a 100 % accuracy rate in bearing fault diagnosis experiments. This performance significantly surpasses that of competing methods. Moreover, the validity of the proposed method based on acoustic-vibrational feature fusion is underscored by the markedly improved accuracy across all models, with nearly all surpassing 99 %. This result not only underscores the exceptional performance of CPO-VMD in the decomposition process, where its enhanced modal decomposition capability facilitates more precise signal characterization for feature extraction, but also emphasizes the comprehensive benefits of acoustic and vibration data fusion in capturing fault features. The integration of acoustic and vibration signals notably expands the dimensionality of fault features, enabling a more effective capture of weak or complex signals that emerge during faults, which significantly boosts the model’s sensitivity to various fault modes.

In practical manufacturing environments, complex operating conditions often lead to deviations between the acquired signals and actual circumstances. Despite the implementation of multiple noise reduction measures in this study, it has not been possible to completely eliminate these interferences, as evidenced by the fluctuations observed in the processed feature data. Consequently, Gaussian white noise was additionally introduced to the smoothed feature vectors, with noise levels set at −4 dB, −6 dB, and −8 dB respectively.

All algorithms compared in this study processed both the noise-treated and untreated datasets following the same procedure. Building on this, an evaluation of methods was conducted under three different noise conditions for fault diagnosis of vibration signals, acoustic signals, and integrated acoustic-vibration signals, encompassing a total of 45 experimental groups. Each group of experiments was repeated ten times to verify the stability and reliability of the experimental results, with the average fault identification accuracy from all trials serving as the final criterion for assessment.

**Fig. 13:**
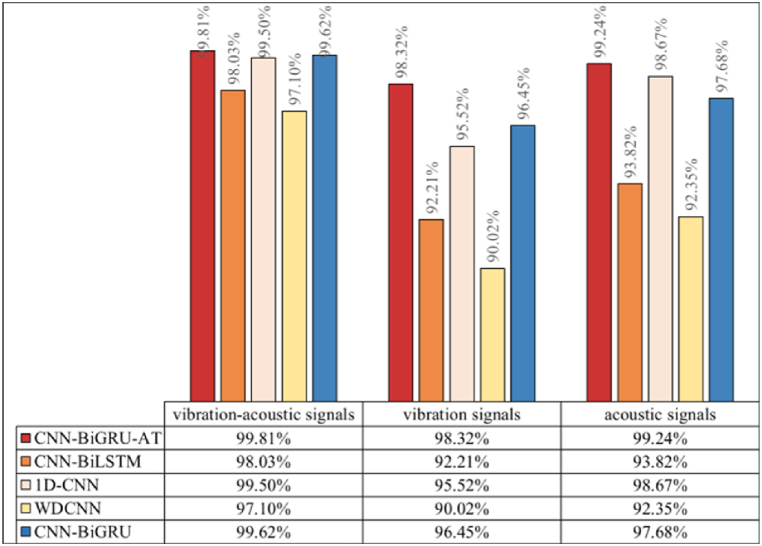
Accuracy of different models (SNR = −4dB).

**Fig. 14:**
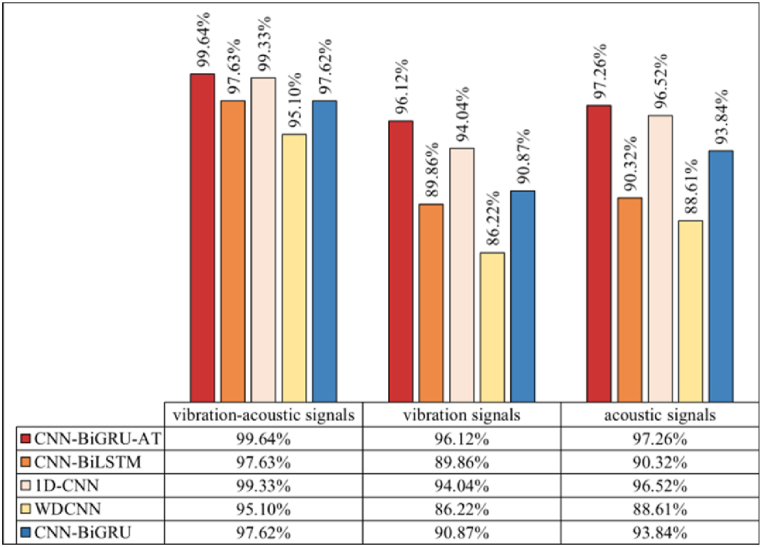
Accuracy of different models (SNR = −6dB).

**Fig. 15:**
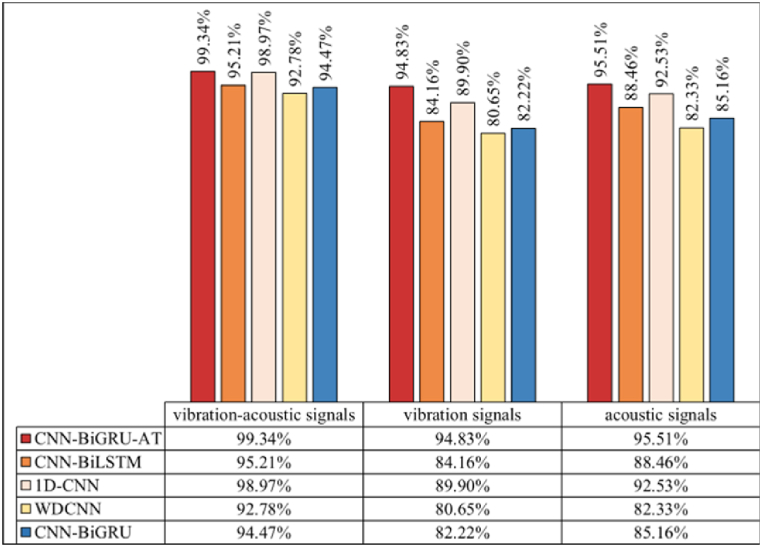
Accuracy of different models (SNR = −8dB).

The results of fault diagnosis under varying noise levels using different methods are depicted in Fig. 13, Fig. 14, Fig. 15. It is observed that the fault identification accuracy of all methods decreases with the increase in noise components. However, the model proposed in this study consistently outperforms comparative methods across three noise environments. Analysis reveals that: (1) As illustrated by Fig. 10, the introduction of artificial noise leads to a reduction in predictive accuracy for all models. When acoustic-vibration signals with “SNR = −4 dB,” “SNR = −6 dB,” and “SNR = −8 dB”are used as inputs, the proposed algorithm achieves accuracies of 99.81 %, 99.64 %, and 99.34 % respectively. In contrast, using raw vibration signals at the same SNRs results in accuracies of 98.32 %, 96.12 %, and 94.83 %, while raw acoustic signals achieve 99.24 %, 97.26 %, and 95.51 %. This suggests that the complementary nature of vibration-acoustic features can provide more comprehensive information in low SNR environments, significantly enhancing the model’s noise resistance and improving fault identification accuracy. (2) As noise levels increase, the diagnostic performance of algorithms based on single-modal sensors shows a noticeable degradation. Background noise directly impacts fault identification accuracy. For instance, the average accuracies of 1D-CNN based on vibration signals at “SNR = −4 dB” and “SNR = −8 dB” are 95.52 % and 89.90 %, a decline of 5.62 percentage points, while those of 1D-CNN based on acoustic signals are 98.67 % and 92.53 %, decreasing by 6.14 percentage points. CNN-BiGRU based on vibration signals achieves average accuracies of 96.45 % and 82.22 %, falling by 14.23 percentage points, and CNN-BiGRU based on acoustic signals records 97.68 % and 85.16 %, dropping by 12.52 percentage points. Conversely, CNN-BiGRU-AT based on vibration signals maintains average accuracies of 98.32 % and 94.83 %, a decrease of 3.49 percentage points, and based on acoustic signals, it records 99.24 % and 95.51 %, decreasing by 3.73 percentage points. These findings demonstrate that the introduction of the self-attention mechanism enables the CNN-BiGRU algorithm to exhibit a lower rate of performance degradation when processing single-modal input signals compared to the other comparative algorithms. This is attributed to the self-attention layer, which provides additional contextual awareness, enhancing the overall stability and reliability of the model in interpreting noisy signals. It is this in-depth analysis and optimization of feature layers that allow the CNN-BiGRU-AT algorithm to maintain high recognition accuracy under various testing conditions, particularly excelling under extreme noise conditions.

## 5 Conclusion

This study introduces a new bearing fault diagnosis method by optimizing the VMD algorithm, which adaptively adjusts the number of modes and the penalty factor. This optimization addresses the traditional reliance on experiential parameter settings and significantly boosts convergence speed, accuracy, and fault feature extraction. Concurrently, the CNN-BiGRU-AT model, by fusing acoustic and vibration signals, achieves accurate fault diagnosis and classification of rolling bearings, with a fault identification rate of 100%—a marked improvement over methods using single-modal sensors. This multimodal approach not only enriches the feature set but also demonstrates robustness and reduced performance degradation under extreme noise conditions, enhanced by a self-attention mechanism, making it well-suited for diverse noise-impacted diagnostic scenarios.

Looking ahead, the potential applications of this multimodal fusion technique are extensive, with plans to broaden its use across various rotating equipment diagnostics. Anticipated further investigations into vibration and acoustic signal fusion strategies are expected to refine diagnostic effectiveness significantly. These advancements aim to provide a more efficient and precise tool for mechanical equipment health monitoring, contributing to the stable and reliable operation of industrial systems. This approach highlights a significant step forward in the integration of machine learning techniques with traditional diagnostic methods, offering a promising outlook for the future of industrial maintenance.

## CRediT authorship contribution statement

**Minyuan Jiang:** Writing – original draft, Visualization, Validation, Supervision, Software, Methodology, Data curation, Conceptualization. **Min Luo:** Writing – review & editing, Funding acquisition. **Chaoyong Zhang:** Writing – review & editing, Funding acquisition, Formal analysis. **Min Shu:** Project administration, Investigation. **Guohao Sun:** Project administration.

## Declaration of competing interest

The authors declare the following financial interests/personal relationships which may be considered as potential competing interests: Luo Min reports article publishing charges, equipment, drugs, or supplies, and writing assistance were provided by Hubei University of Automotive Technology. If there are other authors, they declare that they have no known competing financial interests or personal relationships that could have appeared to influence the work reported in this paper.
